# Cerebrovascular co-pathology and cholinergic white matter pathways along the Lewy body continuum

**DOI:** 10.1093/braincomms/fcaf173

**Published:** 2025-05-06

**Authors:** Cene Jerele, Antonios Tzortzakakis, Milan Nemy, Anna Rennie, Javier Arranz, Victor Montal, Alexandre Bejanin, Dag Aarsland, Eric Westman, Juan Fortea, Alberto Lleó, Daniel Alcolea, Milica G Kramberger, Daniel Ferreira

**Affiliations:** Faculty of Medicine, University of Ljubljana, 1000 Ljubljana, Slovenia; Division of Clinical Geriatrics, Center for Alzheimer Research, Department of Neurobiology, Care Sciences and Society, Karolinska Institute, 171 77 Stockholm, Sweden; Division of Radiology, Department for Clinical Science, Intervention and Technology (CLINTEC), Karolinska Institutet, 141 86 Stockholm, Sweden; Medical Radiation Physics and Nuclear Medicine, Section for Nuclear Medicine, Karolinska University Hospital, Huddinge, 14 186 Stockholm, Sweden; Division of Clinical Geriatrics, Center for Alzheimer Research, Department of Neurobiology, Care Sciences and Society, Karolinska Institute, 171 77 Stockholm, Sweden; Department of Biomedical Engineering and Assistive Technology, Czech Institute of Informatics, Robotics and Cybernetics, Czech Technical University in Prague, 160 00 Prague, Czech Republic; Division of Clinical Geriatrics, Center for Alzheimer Research, Department of Neurobiology, Care Sciences and Society, Karolinska Institute, 171 77 Stockholm, Sweden; Department of Neurology, Hospital de la Santa Creu i Sant Pau, Biomedical Research Institute Sant Pau, Universitat Autònoma de Barcelona, 08025 Barcelona, Spain; Center of Biomedical Investigation Network for Neurodegenerative Diseases (CIBERNED), 28029 Madrid, Spain; Department of Neurology, Hospital de la Santa Creu i Sant Pau, Biomedical Research Institute Sant Pau, Universitat Autònoma de Barcelona, 08025 Barcelona, Spain; Center of Biomedical Investigation Network for Neurodegenerative Diseases (CIBERNED), 28029 Madrid, Spain; Department of Neurology, Hospital de la Santa Creu i Sant Pau, Biomedical Research Institute Sant Pau, Universitat Autònoma de Barcelona, 08025 Barcelona, Spain; Center of Biomedical Investigation Network for Neurodegenerative Diseases (CIBERNED), 28029 Madrid, Spain; Department of Old Age Psychiatry, Institute of Psychiatry, Psychology and Neuroscience, King's College London, SE5 8AB London, UK; Centre for Age-Related Medicine, Stavanger University Hospital, 4068 Stavanger, Norway; Division of Clinical Geriatrics, Center for Alzheimer Research, Department of Neurobiology, Care Sciences and Society, Karolinska Institute, 171 77 Stockholm, Sweden; Department of Neuroimaging, Centre for Neuroimaging Sciences, Institute of Psychiatry, Psychology, and Neuroscience, King's College London, SE5 8AF London, UK; Department of Neurology, Hospital de la Santa Creu i Sant Pau, Biomedical Research Institute Sant Pau, Universitat Autònoma de Barcelona, 08025 Barcelona, Spain; Center of Biomedical Investigation Network for Neurodegenerative Diseases (CIBERNED), 28029 Madrid, Spain; Department of Neurology, Hospital de la Santa Creu i Sant Pau, Biomedical Research Institute Sant Pau, Universitat Autònoma de Barcelona, 08025 Barcelona, Spain; Center of Biomedical Investigation Network for Neurodegenerative Diseases (CIBERNED), 28029 Madrid, Spain; Department of Neurology, Hospital de la Santa Creu i Sant Pau, Biomedical Research Institute Sant Pau, Universitat Autònoma de Barcelona, 08025 Barcelona, Spain; Center of Biomedical Investigation Network for Neurodegenerative Diseases (CIBERNED), 28029 Madrid, Spain; Faculty of Medicine, University of Ljubljana, 1000 Ljubljana, Slovenia; Division of Clinical Geriatrics, Center for Alzheimer Research, Department of Neurobiology, Care Sciences and Society, Karolinska Institute, 171 77 Stockholm, Sweden; Department of Neurology, University Medical Centre Ljubljana, 1000 Ljubljana, Slovenia; Division of Clinical Geriatrics, Center for Alzheimer Research, Department of Neurobiology, Care Sciences and Society, Karolinska Institute, 171 77 Stockholm, Sweden; Facultad de Ciencias de la Salud, Universidad Fernando Pessoa Canarias, 35450 Las Palmas, Spain

**Keywords:** dementia with Lewy bodies, cholinergic white matter pathways, CHIPS, white matter signal abnormalities

## Abstract

Dementia with Lewy bodies often presents with cholinergic degeneration and varying degrees of cerebrovascular disease. There is a lack of radiological methods for evaluating cholinergic degeneration in dementia with Lewy bodies. We investigated the potential of the Cholinergic Pathway Hyperintensities Scale (CHIPS) in identifying cerebrovascular disease–related disruptions in cholinergic white matter pathways, offering a practical and accessible method for assessing cholinergic integrity in neurodegenerative diseases. We assessed the associations of CHIPS with regional brain atrophy, Alzheimer’s disease co-pathology and clinical phenotype. Additionally, we compared its diagnostic performance to that of other manual and automated evaluation methods. We included 82 individuals (41 patients in the Lewy body continuum with either probable dementia with Lewy bodies or mild cognitive impairment with Lewy bodies, and 41 healthy controls) from the Sant Pau Initiative on Neurodegeneration cohort. We used CHIPS to assess cholinergic white matter signal abnormalities (WMSA) on MRI, while tractography mean diffusivity provided a complementary measure of cholinergic WMSA. For global WMSA evaluation, we used the Fazekas scale and FreeSurfer. CHIPS successfully identified cerebrovascular disease–related disruptions in cholinergic white matter pathways, as evidenced by its association with tractography and global WMSA markers (*P* < 0.005 for all associations). Lewy body patients showed a significantly higher degree of WMSA in the external capsule cholinergic pathway despite no significant differences in global WMSA compared to controls. CHIPS score in the posterior external capsule and the mean diffusivity in the external capsule and cingulum exceeded the threshold for an optimal biomarker (sensitivity and specificity values above 80%) in discriminating Lewy body patients from controls. Furthermore, higher CHIPS scores, Fazekas scale and tractography mean diffusivity were associated with more pronounced frontal atrophy in Lewy body patients but not in controls. No associations were found for the four WMSA and integrity methods with the core clinical features, clinical or cognitive measures, or CSF biomarkers. In conclusion, cholinergic WMSA were more pronounced in Lewy body patients compared to healthy controls, independently of global WMSA. Our findings indicate that cerebrovascular disease-related disruptions in cholinergic white matter may be linked to frontal atrophy in Lewy body patients. Clinically, we demonstrate the potential of CHIPS to assess cholinergic WMSA using widely available MRI sequences. Our data suggest cerebrovascular disease co-pathology could drive the cholinergic degeneration in Lewy body patients, opening opportunities for therapeutic interventions targeting vascular health from mild cognitive impairment with Lewy bodies through manifest dementia with Lewy bodies.

## Teaser

Jerele *et al*. report that white matter abnormalities in cholinergic pathways, assessed by Cholinergic Pathway Hyperintensities Scale (CHIPS), are more pronounced in Lewy body patients than controls. Abnormalities are associated with frontal atrophy and may be driven by cerebrovascular disease. CHIPS is useful in assessment of cholinergic white matter abnormalities.

## Graphical Abstract

**Graphical Abstract:**
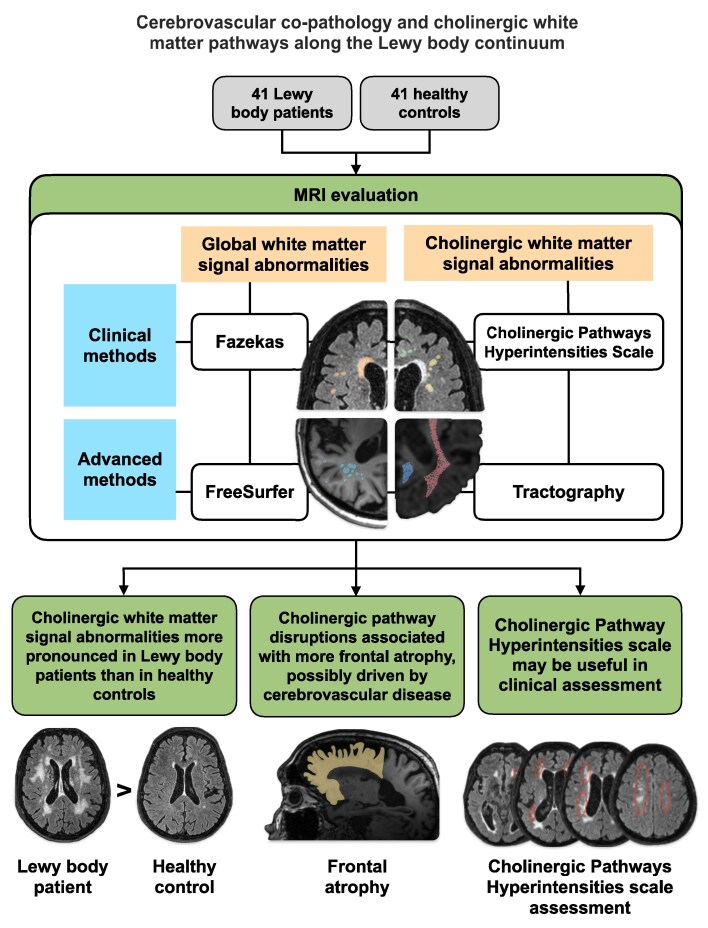

## Introduction

Dementia with Lewy Bodies (DLB) is the second most common cause of neurodegenerative dementia and often presents with cerebrovascular disease (CVD) co-pathology, which contributes to both neurodegeneration and clinical symptoms.^1-6^ White matter signal abnormalities (WMSAs) on magnetic resonance imaging (MRI) are a key marker of CVD and their extent and localization may influence cognitive function. In particular, WMSA affecting cholinergic white matter pathways play a crucial role in cognition across neurodegenerative diseases such as Alzheimer’s disease, vascular dementia and Parkinson’s disease with dementia.^7-9^ While some studies suggest that WMSA are more severe in DLB than in Parkinson’s disease with dementia, the relationship between WMSA and the cholinergic system in DLB remains poorly understood.^10,11^

Cholinergic pathways are affected early in DLB^12^ and their integrity assessed with probabilistic tractography is linked with global cognition and attention in DLB.^13,14^ However, probabilistic tractography is a complex MRI technique that is not widely available in the clinical radiological evaluation of dementia. A simpler, more accessible method for evaluating cholinergic white matter pathways integrity in DLB and other dementias is needed.

The Cholinergic Pathway Hyperintensities Scale (CHIPS) is a semiquantitative visual rating scale for the manual assessment of cholinergic pathways on MRI.^15^ CHIPS has previously been used in individuals with Alzheimer’s and Parkinson's disease.^16-20^ The only previous study using CHIPS in DLB patients focused on comparing the burden of white matter hyperintensities in cholinergic pathways across different neurodegenerative disorders.^19^ However, its ability to detect CVD-related disruptions in cholinergic pathways in DLB patients remains unexplored.

The first aim of this study was to investigate the association of CHIPS with the integrity of cholinergic white matter pathways from tractography as well as with other common MRI markers of WMSA. To address this aim, we mapped cholinergic WMSA using CHIPS (manual rating) and tractography (automated research-oriented measure) and global WMSA using the Fazekas scale (manual rating) and FreeSurfer (automated research-oriented measure). This allowed us to compare CHIPS with an advanced cholinergic white matter integrity measure and global WMSA markers. We compared patient and control groups and investigated associations among measures within groups. Additionally, we calculated sensitivity and specificity values of CHIPS and the other MRI markers to evaluate diagnostic performance for the discrimination between patients and controls. To gain further mechanistic understanding, the second aim of this study was to investigate associations of CHIPS and the other MRI markers with regional brain atrophy, CSF biomarkers of Alzheimer’s disease co-pathology, core clinical features of DLB, and cognitive measures.

## Methods

### Study population

We included 41 patients with a clinical diagnosis of either probable DLB (17 patients) or MCI-LB (24 patients), combined into a single group representing the LB continuum, for statistical analysis. All LB patients were obtained from the Sant Pau Initiative on Neurodegeneration (SPIN) cohort.^21^ For our current study, inclusion criteria for LB patients were consecutive cases assessed between June 2013 and December 2017 at the Sant Pau Memory Unit (Barcelona, Spain), with at least one MRI study and CSF Alzheimer’s disease biomarkers available. Specific details on the evaluation protocol are described elsewhere.^21^ In brief, all individuals enrolled in the SPIN cohort underwent evaluation by neurologists with expertise in neurodegenerative diseases using an extensive neurological and neuropsychological protocol. Although patients were clinically evaluated before the publication of the latest diagnostic criteria, a retrospective review confirmed that all included individuals met the criteria set forth by the DLB Consortium^22^ for DLB and the criteria published by McKeith *et al*.^23^ for MCI-LB. The distinction between MCI-LB and Parkinson's disease with MCI was made following the 1-year rule, as recommended in the same publication.^23^ Core clinical features of parkinsonism, cognitive fluctuations, visual hallucinations and probable REM sleep behaviour disorder were evaluated as part of a comprehensive neurological assessment conducted by experienced neurologists specialized in neurodegenerative diseases, based on the criteria from the DLB consortium.^22^ Patients also provided MRI and CSF samples.

Moreover, 41 healthy individuals from the SPIN cohort were selected as the control group. From the cohort of 89 healthy individuals (SPIN cohort), we selected the oldest 41 individuals as we had 41 LB patients, aiming to match the age distribution of the controls with that of LB patients as closely as possible. Control individuals underwent a thorough medical history review, physical examination and a standard neuropsychological evaluation as previously described.^21^ To be recognized as cognitively unimpaired, control individuals had to meet the criteria of having no subjective cognitive complaints, a mini-mental state examination (MMSE) score of 27–30, a clinical dementia rating (CDR) global score of 0, an free and cued selective reminding test (FCSRT) total immediate adjusted-score of ≥7 and no impairments in daily living activities. Additional details about the inclusion/exclusion criteria and neuropsychological tests used in the SPIN cohort are provided in previous publications.^21^

The study was approved by the local Ethics Committee in Hospital Sant Pau (Barcelona, Spain), following the ethical standards recommended by the Helsinki Declaration. All study individuals provided written informed consent.

### MRI scanning

A high-resolution 3D T1-weighted magnetization-prepared rapid gradient echo (MPRAGE) sequence, a fluid-attenuated inversion recovery (FLAIR) sequence and a diffusion-weighted imaging (DWI) sequence were acquired for each individual at the Hospital de la Santa Creu i Sant Pau (Barcelona, Spain). All 41 LB patients and 41 controls underwent MRI scanning using the 3T Philips Achieva scanner (Philips Healthcare). The scanning parameters are provided in the Supplementary Table 1.

### Global and regional manual measures of WMSA

The manual measures included the Fazekas scale for global WMSA and CHIPS for regional cholinergic WMSA.

**Figure 1:**
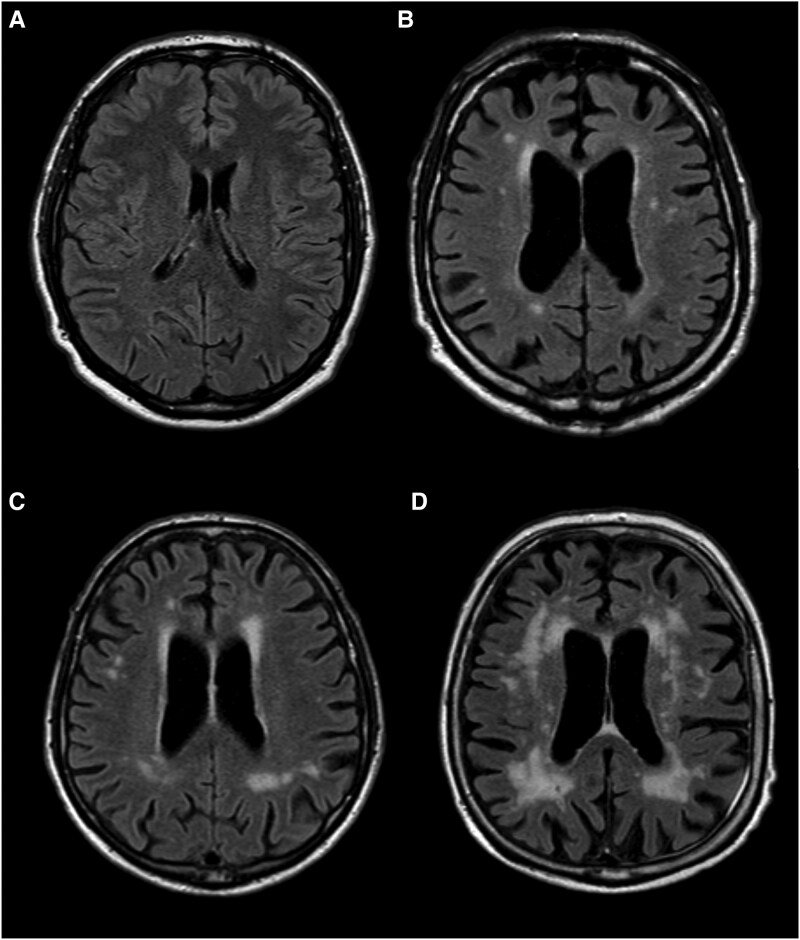
**Visual representation of Fazekas scale stages (0–3) for white matter signal abnormalities (WMSA).** This figure illustrates the four stages of the Fazekas scale, a widely used visual rating scale for assessing WMSA on MRI. (**A**) Fazekas 0—No WMSA visible. (**B**) Fazekas 1—Mild periventricular caps and punctated foci in the deep white matter. (**C**) Fazekas 2—Moderate WMSA with a beginning confluence of foci. (**D**) Fazekas 3—Severe WMSA, with large confluent lesions extending throughout the white matter.

The Fazekas scale was assessed on FLAIR images using the modified Fazekas scale for periventricular and deep white matter regions (Fig. 1).^24^ For statistical analyses, Fazekas scores of 0 or 1 were classified as a low-WMSA group (low Fazekas group), and Fazekas scores of 2 or 3 were classified as a high-WMSA group (high Fazekas group), following the approach of previous studies.^10^

**Figure 2:**
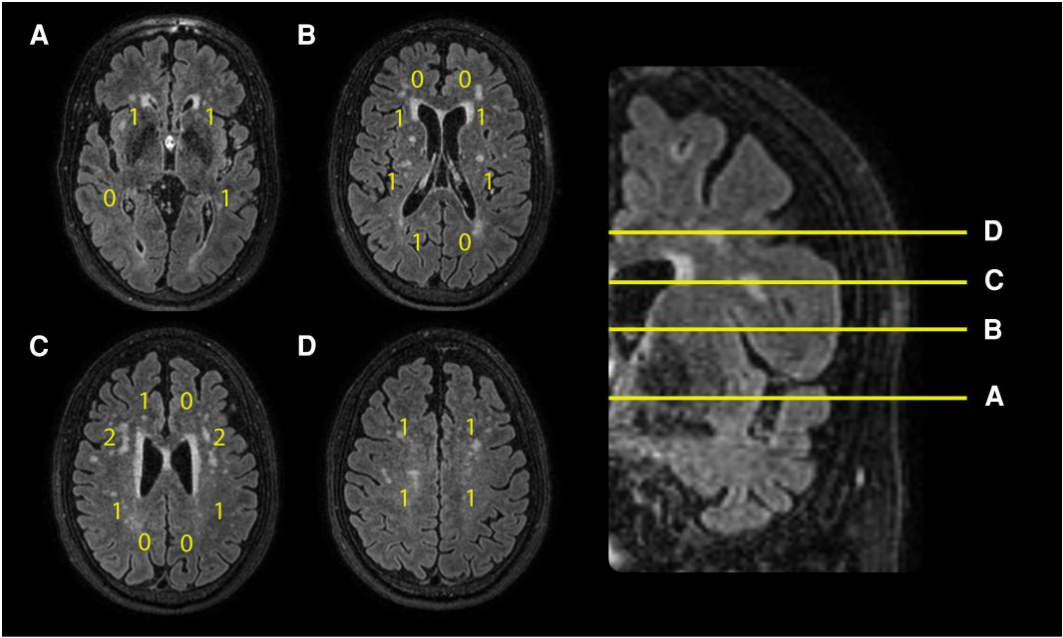
**An example of CHIPS scoring illustrated on FLAIR sequence MRI images.** To perform the CHIPS ratings, major anatomical landmarks on four index slices in the axial plane were selected: low external capsule (**A**), high external capsule (**B**), corona radiata (**C**) and centrum semiovale (**D**). Examples of CHIPS scores are shown in **A–D**. (**A**) Low external capsule: anterior (right = 1, left = 1, factor = ×4, total = 8); posterior (right = 0, left = 1, factor = ×4, total = 4). (**B**) High external capsule: anterior (right = 1, left = 1, factor = ×3, total = 6); posterior (right = 1, left = 1, factor = ×3, total = 6); cingulate (right = 1, left = 0, factor = ×4, total = 4). (**C**) Corona radiata: anterior (right = 2, left = 2, factor = ×2, total = 8); posterior (right = 1, left = 1, factor = ×2, total = 4); cingulate (right = 1, left = 0, factor = ×1, total = 1). (**D**) Centrum semiovale: anterior (right = 1, left = 1, factor = ×1, total = 2); posterior (right = 1, left = 1, factor = ×1, total = 2). The total CHIPS score is 45. (**E**) Shows respective levels of slices **A–D** on a coronal FLAIR MRI. CHIPS, Cholinergic Pathways Hyperintensities Score; FLAIR, fluid-attenuated inversion recovery; NBM, Nucleus basalis of Meynert.

CHIPS was assessed on axial sections of FLAIR images as previously described by Bocti *et al*.^15^ CHIPS is based on anatomical landmarks from immunohistochemical studies and evaluates WMSA in the medial (cingulum) and lateral (external capsule) cholinergic pathways. MRIs are rated at four different standardized axial slices including those with the third ventricle and claustrum (low external capsule), the top of the sylvian fissure (high external capsule), the high lateral ventricle (corona radiata) and the top of the corpus callosum (centrum semiovale), with separate ratings for anterior and posterior regions at each level (Fig. 2). Each region is rated using a three-point system (0 = normal; 1 = <50% involvement; 2 ≥ 50% involvement). A factor of 4 is attributed to the lower standardized axial level with linearly decreasing factors for each successive higher level, to account for decreasing cholinergic fibre density. Details on regions scoring and corresponding factors are shown in Supplementary Table 2. The maximum possible score is 50 for each hemisphere, yielding a total CHIPS score of 100 points. An example of CHIPS scoring illustrated on FLAIR MRI is presented in Fig. 2. In this study, we used the total CHIPS score as well as CHIPS subregions anterior external capsule, posterior external capsule, and cingulum for statistical analyses. CHIPS anterior and posterior external capsule scores were calculated by summing the left and right hemisphere scores for the respective anterior and posterior regions on each evaluated slice. The final score was obtained by adding the summed scores across all four slices. To evaluate the potential of CHIPS in identifying CVD-related disruptions, we examined its associations with tractography-based measures of cholinergic integrity, as well as global WMSA markers (Fazekas and FreeSurfer WMSA volume). Tractography is a well-established method for assessing cholinergic white matter integrity, and Fazekas and WMSA volume are surrogate markers of CVD, allowing us to compare CHIPS performance against widely accepted reference measures.^13,14,25^

All images were rated by an experienced neuroradiologist (A.T.) and a radiology resident (C.J.), to ensure interrater agreement. Both raters assessed the images with CHIPS and Fazekas scales. The raters performed the ratings separately using the same hardware and software and were blinded to any information about the study individuals. In cases of disagreement between the two raters, a consensus for the final score was reached through discussion. Interrater agreement between the two radiologists was calculated and expressed as quadratic weighted Kappa coefficients. The benchmark proposed by Landis *et al*.^26^ was used to interpret the level of agreement between the raters. Kappa scores close to 0.0 were interpreted as poor agreement, 0.01–0.20 as slight agreement, 0.21–0.40 as fair agreement, 0.41–0.60 as moderate agreement, 0.61–0.80 as substantial agreement and 0.81–1.0 as almost perfect agreement.

### Automated segmentation of global WMSA assessed with FreeSurfer

We used FreeSurfer v7.3.1 Image Analysis software to segment white matter hypointensities on T1-weighted images as a volumetric marker of WMSA.^27^ Previous findings revealed that hypointense WMSA might represent necrotic damage closer to accumulated cerebrovascular pathology, whereas hyperintense WMLs might also represent acute damage including peri-inflammatory processes.^28,29^ Since our current study focused on cerebrovascular disease biomarkers, we decided to use hypointense WMSA in our statistical analysis. Additionally, we previously demonstrated the significant and strong association between white matter hypointensities on T1-weighted images and hyperintensities on T2/FLAIR sequences.^30^ Regarding WMSA segmentation, FreeSurfer employs an algorithm that assigns a label to each voxel based on probabilistic local and intensity-related information that is automatically estimated from a built-in training dataset comprising 41 manually segmented images (surfer.nmr.mgh.harvard.edu/fswiki/AtlasSubjects^27^). This approach has exhibited good sensitivity in evaluating white matter alterations both in healthy individuals and patients with Alzheimer’s disease.^31,32^ Additionally, FreeSurfer software was used to estimate the total intracranial volume (TIV) for each individual. Data were organized and processed through the TheHiveDB system.^33^

### Tractography analysis of cholinergic white matter pathways

The procedure for tractography analysis has been described in a previous study.^25^ Briefly, the diffusion-weighted imaging data were preprocessed using FSL (FMRIB Software Library).^34^ This included the removal of non-brain tissue and correction for EPI distortion, eddy currents and head motion.^35,36^ Probabilistic tracking was performed by repeating 5000 random samples from each of the voxels within a mask of the nucleus basalis of Meynert (NBM).^37^ Our study focuses on cholinergic dysfunction in LB patients, particularly, as a potential contributor to the cognitive impairment and clinical phenotype in LB patients. Therefore, we targeted the Ch4 region (NBM and substantia innominata) rather than the medial septal and diagonal band nuclei, which primarily project to the hippocampus and olfactory bulb and are less related to cognitive impairment. The NBM region of interest (ROI) was based on a cytoarchitectonic map of basal forebrain cholinergic nuclei in MNI space, derived from combined histology and MRI of a post-mortem brain, as described by Kilimann *et al*.^38^ We obtained the NBM ROI by combining the anterior lateral, intermediate, and posterior regions of the Ch4 region of the basal forebrain mask. This ROI mask was registered to each subject’s individual space using the inverse of the registration parameters from T1-MNI space, followed by registration to native diffusion space, utilizing the non-linear SyN registration algorithm in Advanced Normalization Tools (ANTs, http://stnava.github.io/ANTs/).

**Figure 3:**
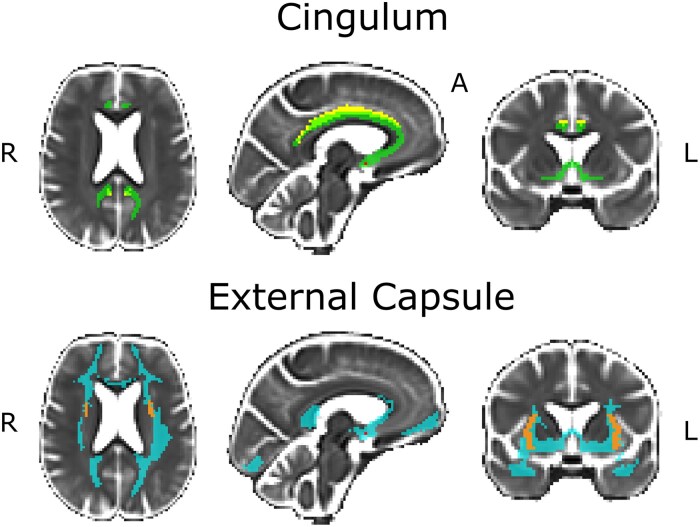
**Tractography analysis of cholinergic white matter pathways.** Cholinergic white matter pathways passing through the cingulum and external capsule used in tractography analysis. (Cingulum) Yellow for the cingulum mask, green for the pathway passing through the cingulum. (External Capsule) Orange for the external capsule mask, blue for the pathway passing through the external capsule. LEC, lower external capsule; HEC, higher external capsule; CR, corona radiata; CSO, Centrum semiovale; R, right; A, anterior; L, left.

Diffusion tracking was performed in a constrained manner using FSL's probtrackX with default parameters.^37^ Probabilistic tracking was initiated from each voxel within the NBM ROI, with 5000 streamlines per voxel. Only streamlines reaching a voxel in midway cingulum or external capsule ROI masks were retained. This approach aimed to reconstruct the two major NBM cholinergic pathways projecting through the cingulum and the external capsule and filter out potential false positives (non-cholinergic streamlines; Fig. 3).^39,40^ Next, an unbiased template was created based on preprocessed images acquired without diffusion weighting (b0 images), and the cingulum and external capsule cholinergic pathways of all study individuals were non-linearly warped into the space of the unbiased template. Finally, pathway-specific binary masks were created by considering all the individual warped tracts and retaining only the voxels that were present in at least 50% of the cases. The 50% group threshold was chosen by visual inspection so that the resulting pathways were extensive yet still specific, as in our previous publication.^25^ To prevent WMSA affecting the tractography results, we built the cholinergic segmentation model using only healthy individuals, who have significantly lower WMSA volumes than patients with cognitive impairment. This approach helps reduce potential bias.

To characterize the microstructure properties of the tracked cholinergic pathways, we extracted the index of mean diffusivity (MD) from the diffusion tensor model, consistent with previous studies using this tractography method.^13,14,25,41,42^ We selected the MD index because it has been shown to precede changes in other diffusion tensor imaging indexes such as fractional anisotropy, and in grey matter volume.^43-45^ Additionally, MD is less susceptible than fractional anisotropy to issues related to different fibre populations within individual voxels, addressing the crossing fibres problem.

### Measures of regional brain atrophy

To assess the degree of atrophy on MRI, we used AVRA (Automatic Visual Ratings of Atrophy), an artificial intelligence-based method trained on ratings from an experienced neuroradiologist.^46^ AVRA has been shown to mimic the neuroradiologist’s rating procedure and achieves similar levels of inter-rater agreement to that obtained between two experienced neuroradiologists, with the advantage that intra-rater AVRA agreements are always 100%.^46^ AVRA provides fast and automatic ratings for the Scheltens’ scale of medial temporal atrophy (MTA), the frontal subscale of Pasquier’s Global Cortical Atrophy (GCA-F) scale and the Koedam’s scale of Posterior Atrophy (PA).^46^ Another advantage of AVRA is that it provides both continuous and categorical measures of atrophy. We applied previously published clinical cut-offs to further classify AVRA scores as normal or abnormal.^47^ Specifically, MTA scores ≥1.5, ≥1.5, ≥2 and ≥2.5 were considered abnormal for the respective age ranges of 45–64, 65–74, 75–84 and 85–94 years. For PA and GCA-F, a score of ≥1 was deemed abnormal regardless of the age range.^47^

### CSF biomarkers

Lumbar puncture and CSF analysis procedures are detailed in previous publications.^21^ Core Alzheimer’s disease biomarkers were measured using the Lumipulse G β-Amyloid 1-42, β-Amyloid 1-40, Total Tau and pTau 181 assays on LUMIPULSE G600II automated platform (Fujirebio).^48^ Cut-off values for these core Alzheimer’s disease biomarkers were based on previously published 18F-Florbetapir PET optimized cut-off values.^48^

### Statistical analysis

Demographic and clinical characteristics were reported as means and standard deviations for continuous variables and as counts and percentages for categorical variables.

Group differences between LB patients and healthy controls were assessed using independent-sample *t*-tests, ANCOVA (for comparisons adjusting for covariates such as age and education), and Mann–Whitney U-tests for non-normally distributed variables. Chi-squared tests were used to assess differences in categorical variables.

Within-group paired associations among MRI markers (CHIPS, Fazekas, FreeSurfer WMSA volume, and tractography MD) were assessed using these same statistical tests than for group differences when one of the MRI markers was dichotomous. Otherwise, when both MRI markers were continuous, we used Pearson’s correlations and multiple linear regression for normally distributed variables, and Spearman’s correlations for non-normally distributed variables. Additionally, within-group associations between these MRI markers and regional atrophy, CSF biomarkers, core clinical features, and cognitive measures were examined using Pearson’s correlations and multiple linear regression for normally distributed variables, and Spearman’s correlations for non-normally distributed variables.

Hence, the choice of test depended on the nature of the variables (continuous versus categorical), the number of variables involved (correlation versus multiple regression), and the type of comparison (*t*-test versus ANCOVA for multiple categories). Effect sizes were expressed as Cohen’s d, allowing for direct comparison across all these different statistical tests. Cohen’s d values for group differences were calculated directly from *t*-tests, while for correlations, they were converted using the formula *d* = 2*r*/√(1 − *r*^2^).

Since there was a significant difference in mean age and education between LB patients and healthy controls, comparisons between these groups were adjusted for age and education using ANCOVA. To get a further understating on the effect of age, for some variables of interest ANCOVA was followed by a test for the statistical interaction between diagnostic group and age in the prediction of MRI marker ‘X’. This test enabled assessing whether associations between age and ‘X’ were stronger in LB patients than in healthy controls. Additionally, FreeSurfer WMSA volumes were adjusted for total intracranial volume (TIV) also using ANCOVA.

In within-group analyses, we did not use age as a covariate because age is intrinsically linked to disease progression along the LB continuum, making it a core component of our LB group rather than a confounding variable. Using age as a covariate might obscure disease-specific changes. Instead, we reported associations separately for LB patients and controls. Based on that reporting, findings that were statistically significant in LB but not in controls were interpreted as disease-specific. Findings that were statistically significant in both groups were followed by a test for the statistical interaction between MRI marker ‘X’ and diagnostic group in the prediction of any other measure (MRI marker, CSF biomarker, core clinical feature, cognitive measure). This test enabled demonstrating stronger associations in LB patients beyond and above those found in the healthy population. When no statistically significant interaction was found, findings in the LB group were consider to reflect those found in a healthy population. This approach allows differentiating disease-specific effects from normal aging-related effects.

To investigate diagnostic performance for the discrimination between LB patients and controls, receiver operating characteristic (ROC) curves were generated for CHIPS and other MRI markers. The area under the curve (AUC), along with 95% confidence intervals, cutoff values, sensitivity, and specificity values, were reported. Youden’s *J* statistic was used to determine the cut-off values with the highest sensitivity and specificity.

Statistical significance was set at *P* < 0.05 for all analyses. All statistical analyses were conducted using IBM SPSS Statistics v26 (IBM Corp., Armonk, NY, USA).

## Results

### Cohort characteristics

Individuals’ characteristics are listed in Table 1. The LB patients and control groups were statistically comparable in sex distribution (*P* = 0.269), while the mean age was higher in LB patients than in controls (*P* < 0.001). The data on core clinical features, regional brain atrophy, and CSF biomarkers are shown in Table 2.

**Table 1:** Cohort characteristics

	Lewy body group (*n* = 41)			Healthy controls (*n* = 41)	
	*N*	Mean (SD)/count (%)	*N*	Mean (SD)/Count (%)	*P*-value
Age, years	41	75.9 (5.4)	41	67.6 (6.2)	**<0**.**001**
Min-max		58–85		60–86	
Sex, female	41	19 (46%)	41	24 (59%)	0.269
Education, years	41	9.6 (5.0)	41	15 (4.4)	**<0**.**001**
Visual hallucinations, presence	41	18 (44%)	41	0 (0%)	**<0**.**001**
RBD, presence	41	25 (61%)	41	0 (0%)	**<0**.**001**
Cognitive fluctuations, presence	41	32 (78%)	41	0 (0%)	**<0**.**001**
Parkinsonism, presence	41	40 (98%)	41	0 (0%)	**<0**.**001**
Unified Parkinson's disease rating scale	33	18.8 (10.6)	0		
Geriatric depression scale	38	11.7 (6.3)	0		
Neuropsychiatric inventory total score	24	14.8 (12.3)	29	0.4 (0.9)	**<0**.**001**
Global deterioration scale	41	3.5 (0.6)	0		
Clinical dementia rating, 0; 0.5; 1; 2; 3	38	1 (2.6%); 27 (73.7%); 8 (18.7%); 2 (5.3%); 0 (0%)	37	36 (97.7%); 1 (2.7%); 0 (0%); 0 (0%); 0 (0%)	**<0**.**001**
Mini-mental state examination	40	24.9 (3.6)	40	28.9 (1.1)	**<0**.**001**
Digits span—direct	38	4.5 (1.0)	40	5.3 (0.9)	0.19
Digits span—reverse	38	3.2 (1.0)	40	4.5 (1.1)	0.057
Boston naming Test	36	43.3 (7.7)	40	55.4 (3.4)	**<0**.**001**
Semantic fluency	38	11.3 (4.1)	40	19.6 (4.0)	**<0**.**001**
Phonetic fluency	38	7.7 (4.1)	40	15.8 (5.1)	**<0**.**001**
FCSRT total free recall	37	10.4 (8.4)	40	27.3 (6.2)	**<0**.**001**
FCSRT total recall	37	26.8 (13.6)	40	44.3 (3.5)	**<0**.**001**
Visual objects and space perception battery	38	6.5 (2.8)	40	9.2 (1.2)	**0**.**001**
DAT scan, pathological	26	20 (77%)	0		

Mann–Whitney U-test was used to evaluate differences in age and education between the Lewy body group and healthy controls. The Chi-squared test was used for the evaluation of the differences in sex distribution between the two groups. ANCOVA with age and education as covariates was used for all other comparisons in Table 1. Statistical significance was set at *P* < 0.05 for all analyses (significant results marked in bold).

DAT, dopamine active transporter; FCSRT, Free and Cued Selective Reminding Test; RBD, rapid eye movement sleep behavioural disorder.

**Table 2:** WMSA and CSF biomarkers

		Lewy body group (*n* = 41)		Healthy controls (*n* = 41)	*P*-value
	*N*	Mean (SD)/count (%)	*N*	Mean (SD)/Count (%)	
CHIPS total	41	30.9 (15.9)	41	9.8 (9.3)	**<0**.**001**
CHIPS external capsule	41	28.9 (14.2)	41	9.1 (8.9)	**<0**.**001**
CHIPS external capsule—anterior	41	15.0 (7.6)	41	6.4 (5.6)	**0**.**021**
CHIPS external capsule—posterior	41	13.9 (7.8)	41	2.7 (4.2)	**<0**.**001**
CHIPS cingulum	41	2.0 (2.7)	41	0.7 (1.4)	0.377
High WMSA (Fazekas 2–3)	41	25 (61%)	41	11 (26.8%)	0.126
Freesurfer WMSA	40	5855 (5057)	39	2210 (2185)	0.492
Tractography cingulum	37	0.00109 (0.00011)	37	0.00095 (0.00007)	**0**.**040**
Tractography external capsule	37	0.00129 (0.00011)	37	0.00112 (0.00009)	**0**.**031**
MTA	41	1.61 (0.75)	41	0.65 (0.47)	**<0**.**001**
MTA (% abnormal)	41	39.0	41	5.0	**<0**.**001**
PA	41	0.64 (0.5)	41	0.50 (0.46)	0.741
PA (% abnormal)	41	24.4	41	7.5	0.795
GCA-F	41	0.22 (0.28)	41	0.02 (0.09)	**0**.**012**
GCA-F (% abnormal)	41	2.4	41	0.0	0.888
AB42–40 ratio	40	0.066 (0.03)	31	0.125 (0.19)	0.308
AB42–40 ratio (% abnormal)	40	60%	31	19%	**0**.**048**
Total tau	40	471 (253)	31	361 (260)	0.377
Total tau (% abnormal)	40	43%	31	19%	0.784
Phosphorylated tau	40	77.6 (49.7)	31	55.3 (55.1)	0.300
Phosphorylated tau (% abnormal)	40	50%	31	19%	0.225
AD CSF profile	40	19 (47.5%)	31	4 (12.9%)	**0**.**002**

MTA scores 1.5, 2 and 2.5 were considered abnormal for the respective age ranges 45–74, 75–84 and 85–94 years. A score of equal or larger than 1 was considered abnormal irrespectively of the age range for PA and GCA-F. These cut-offs were based on previously published criteria (Ferreira *et al*.^47^). AB42–40 ratio = Amyloid beta 1–42/Amyloid beta 1–40 ratio. CSF biomarkers were classified as abnormal based on the following cut-offs: tTau > 456 pg/mL, pTau > 63 pg/mL, and Aβ42/Aβ40 < 0.062. Alzheimer’s disease CSF profile was defined as abnormal AB42–40 ratio and abnormal pTau based on previously specified values. All comparisons between Lewy body group and healthy controls in Table 2 were conducted using ANCOVA, with age and education included as covariates. Statistical significance was set at *P* < 0.05 for all analyses (significant results marked in bold). CHIPS, Cholinergic pathways hyperintensities scale; GCA-F, Global cortical atrophy—frontal; MTA, medial temporal atrophy, abnormal > ; PA, parietal atrophy; WMSA, white matter signal abnormalities.

### Interrater agreement

The weighted Kappa for CHIPS was 0.93, while Fazekas’s weighted Kappa was 0.88. Radiologists thus showed almost perfect agreement on both scales.

### Associations with age, sex, and education for selection of covariates

**Figure 4:**
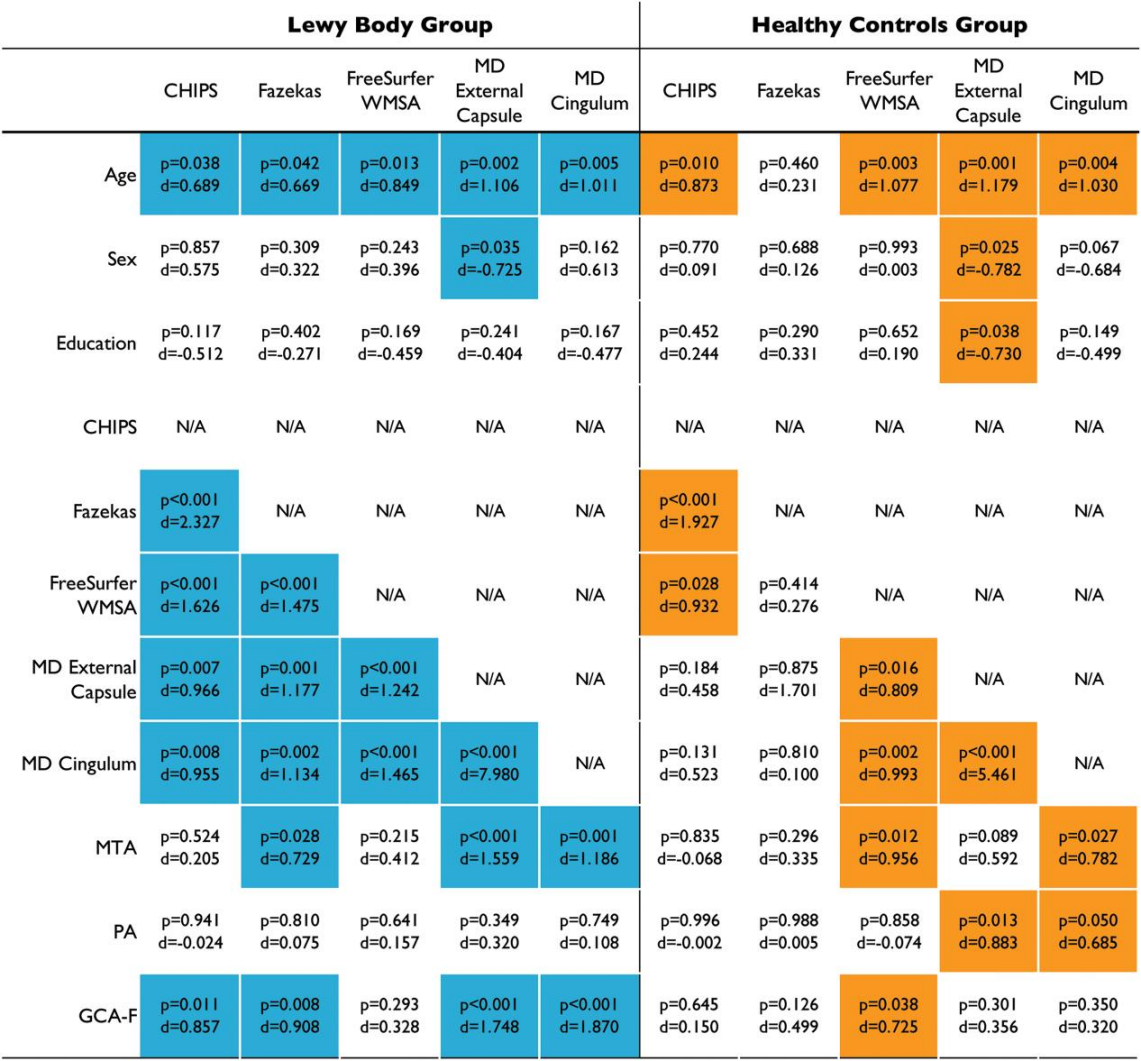
**Summary of findings.** Coloured squares signify a statistically significant association between the two measures. Empty rectangles signify non-significant associations. *P*-values and effects sizes expressed as Cohen’s d are included for variable pairs. Cohen’s d was used to allow for direct comparison across different statistical tests. Statistical significance was set at *P* < 0.05 for all analyses. The actual statistical test in each table cell was chosen based on the nature of the variables (continuous, categorical, normally distributed, non-normally distributed). In particular, we used Pearson’s correlations, independent samples *t*-tests, multiple linear regression and ANCOVA for normally distributed variables. For non-normally distributed variables, we used Spearman’s correlations and the Mann–Whitney U-test. The Chi-square test was used for associations between two categorical variables. N/A, not applicable, used for duplicated variable pairs; CHIPS, Cholinergic Pathways Hyperintensities Score; WMSA, White Matter Signal Abnormalities; MD , mean diffusivity; MTA, medial temporal atrophy; PA, parietal atrophy; GCA-F, Global Cortical Atrophy—Frontal.

We investigated associations with age, sex, and education for selection of covariates in further analyses targeting the main aims of this study. Please see Fig. 4 and Supplementary Table 3 for a summary of findings, *P*-values and effect sizes.

The high Fazekas group had an older age than the low Fazekas group in LB patients (*P* = 0.042). Similarly, higher CHIPS scores were associated with older age in LB patients (*P* = 0.038), and higher CHIPS scores with older age in controls (*P* = 0.010).

A higher FreeSurfer global WMSA volume (TIV adjusted) was associated with a higher age both in LB patients and controls (*P* = 0.002 and *P* = 0.005, respectively).

For cholinergic white matter pathways (tractography), higher age was associated with higher mean diffusivity in the cingulum and external capsule in both LB (*P* = 0.005, *P* = 0.002) and controls (*P* = 0.004, *P* = 0.001). Mean diffusivity in the external capsule was higher in males (*P* = 0.028 in LB, *P* = 0.045 in controls). In controls, higher external capsule diffusivity was associated with fewer years of education (*P* = 0.038).

### Aim 1 part 1—group differences in WMSA markers and white matter cholinergic pathways (tractography)

Based on the findings above, all analyses in this section were adjusted for age and education (and TIV for FreeSurfer WMSA).

LB patients and controls did not differ significantly in the proportion of high Fazekas group (*P* = 0.126) or FreeSurfer global WMSA volume (*P* = 0.492).

In contrast, mean total CHIPS scores were higher in LB patients compared to controls (30.9 ± 15.9 versus 9.8 ± 9.3, *P* < 0.001), including the external capsule (*P* < 0.001) and its anterior (*P* = 0.012) and posterior subregions (*P* < 0.001), while no differences were observed for the cingulum (*P* = 0.377).

**Figure 5:**
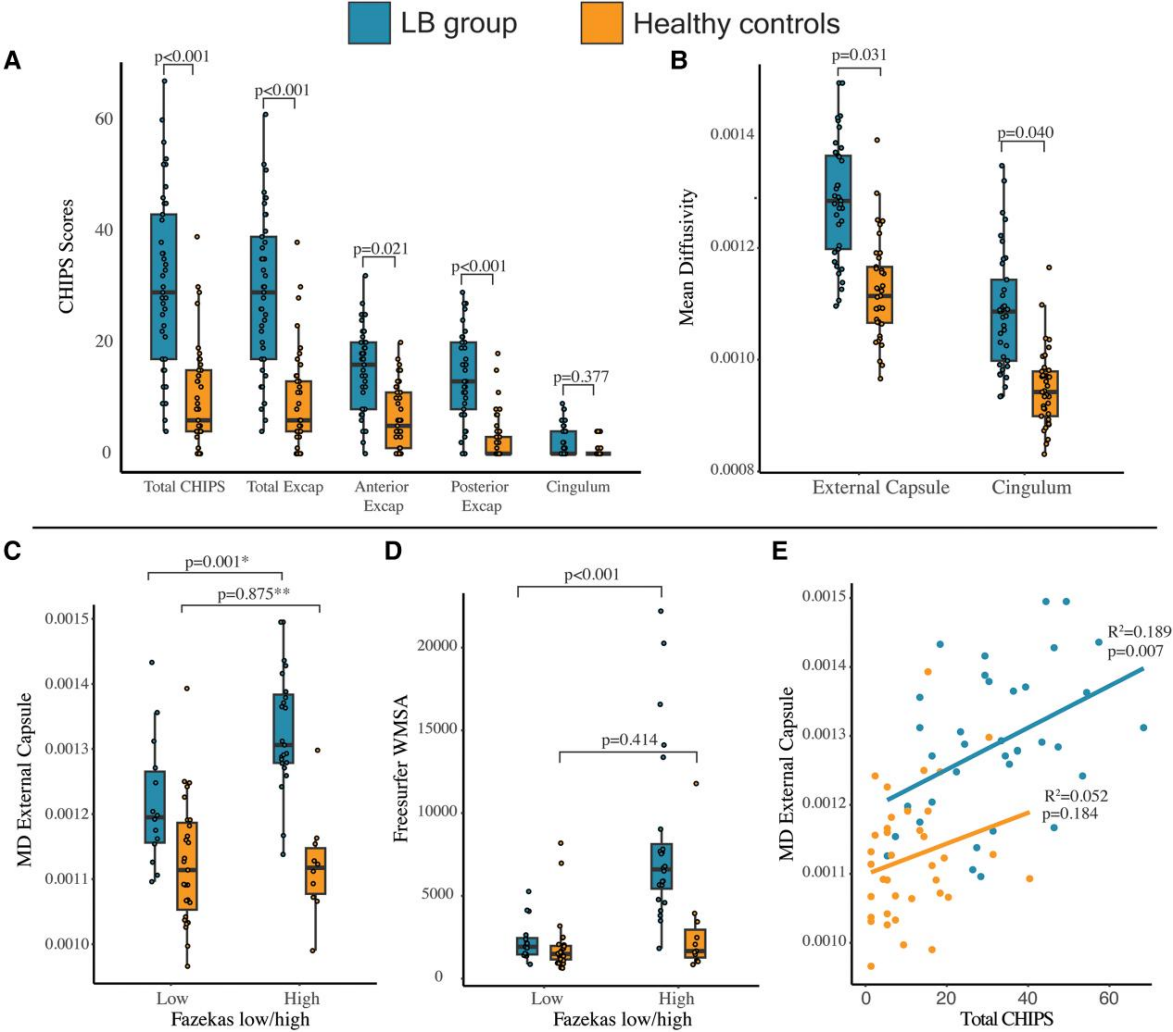
**Group comparison and associations among WMSA measures and white matter cholinergic pathways.** (**A**) CHIPS scores by subdomain, comparison between LB patients and controls. (**B**) Cholinergic white matter pathways passing through the cingulum and external capsule used in tractography analysis, comparison between LB patients and controls. (**C**, **D**, **E**) Association between WMSA markers and cholinergic white matter pathways, by diagnostic group. MD External Capsule depicted in **C**, but similar results were obtained for MD Cingulum as reported in Fig. 4. Statistical tests used: **A** and **B**, ANCOVA (age and education as covariates), **C**, Mann–Whitney U-test; **D**, Multiple linear regression (TIV as a covariate); figure **E**, Spearman correlation with linear regression lines fitted separately for LB and control groups. Bonferroni correction for multiple testing in **C** corresponds with *P = 0.002, and **P = 1.0. Excap, External Capsule; MD, mean diffusivity; CHIPS, Cholinergic pathways hyperintensities scale; LB, Lewy body; WMSA, white matter signal abnormalities.

Additionally, mean diffusivity was higher in LB patients in both the external capsule (*P* = 0.031) and cingulum cholinergic pathways (*P* = 0.040) compared with controls (Fig. 5B). Please see Fig. 4 for the summary of findings.

### Aim 1 part 2—associations among WMSA markers and white matter cholinergic pathways (tractography)

Figure 4 provides a summary of the associations between MRI markers. As it can be observed, there were more significant associations in LB patients than in controls. Both LB patients and controls in the high Fazekas group had higher CHIPS scores (*P* < 0.001 in both). Higher CHIPS scores were associated with higher FreeSurfer global WMSA volume (*P* < 0.001 in LB patients, *P* = 0.028 in controls), with no significant interaction effects (*P* > 0.05).

LB patients in the high Fazekas group had a higher FreeSurfer WMSA volume (*P* < 0.001), while there were no significant differences in controls (*P* = 0.414; Fig. 5D).

In the LB group, higher CHIPS scores and those in the high Fazekas group were associated with a higher mean diffusivity in both the cingulum and external capsule cholinergic pathways (*P* < 0.05). However, in controls, cholinergic pathway mean diffusivity was not associated with CHIPS or Fazekas group (*P* > 0.05; Fig. 5C and E).

Full details are available in the Fig. 4.

### Aim 1 part 3—diagnostic performance of CHIPS and other MRI markers for discriminating LB patients from controls

**Figure 6:**
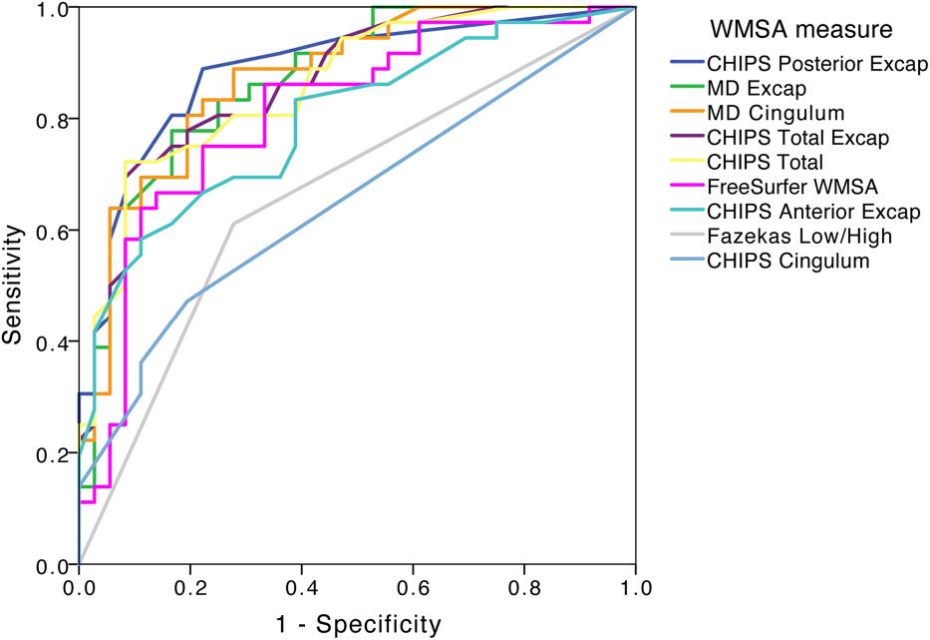
**Diagnostic performance of WMSA measures and tractography analysis in discriminating LB patients from controls: ROC analysis.** CHIPS posterior external capsule (AUC = 0.887, *P* < 0.001); MD external capsule (AUC = 0.873, *P* < 0.001); MD cingulum (AUC = 0.873, *P* < 0.001); CHIPS total external capsule (AUC = 0.868, *P* < 0.001); total CHIPS score (AUC = 0.861, *P* < 0.001); FreeSurfer WMSA (AUC = 0.810, *P* < 0.001); CHIPS anterior external capsule (AUC = 0.797, *P* < 0.001); Fazekas score (AUC = 0.667, *P* = 0.015); CHIPS cingulum (AUC = 0.652, *P* = 0.026). CHIPS, Cholinergic pathways hyperintensities scale; MD, mean diffusivity; Excap, external capsule; WMSA, white matter signal abnormalities; LB, Lewy body.

ROC analysis results are summarized in Fig. 6 and fully detailed in Supplementary Table 4. CHIPS in the posterior external capsule had the highest AUC (0.887), followed by mean diffusivity in the external capsule (AUC = 0.873), total external capsule CHIPS (AUC = 0.868) and total CHIPS score (AUC = 0.861).

The Fazekas scale (AUC = 0.667) and CHIPS in the cingulum (AUC = 0.652) showed the lowest discrimination. FreeSurfer global WMSA volume had an AUC of 0.810, and CHIPS in the anterior external capsule showed an AUC of 0.797.

### Aim 2 part 1—associations with regional brain atrophy

Table 2 presents atrophy scores (MTA, GCA-F, PA) both as continuous values and classified using clinical cut-offs. An abnormal MTA was observed in 39% of LB patients versus 5% of controls, PA in 24.4% versus 7.5% and GCA-F in 2.4% versus 0%. Subsequent analyses were performed on continuous values.

In LB patients, higher CHIPS scores were associated with more frontal atrophy (GCA-F, *P* = 0.011) but not with MTA or PA (*P* > 0.05). No significant associations were found in controls. The high Fazekas group in LB patients presented with more medial temporal (MTA, *P* = 0.023) and more frontal atrophy (GCA-F, *P* = 0.008) but not posterior atrophy (PA, *P* = 0.810).

FreeSurfer global WMSA volume was not associated with atrophy in LB patients (*P* > 0.05) but correlated with MTA (*P* = 0.001) and GCA-F (*P* < 0.001) in controls.

In LB patients, higher mean diffusivity in both external capsule and cingulum cholinergic pathways was associated with more medial temporal (MTA, *P* < 0.001) and frontal atrophy (GCA-F, *P* < 0.001) but not posterior atrophy (PA, *P* > 0.05). In controls, cingulum diffusivity was associated with MTA (*P* = 0.027) and PA (*P* = 0.05), while external capsule diffusivity was associated with PA (*P* = 0.013) but not MTA or GCA-F (*P* > 0.05).

Full details are available in the Fig. 4.

### Aim 2 part 2—associations with CSF biomarkers, clinical features, and cognitive performance

CHIPS, Fazekas, FreeSurfer global WMSA volume and cholinergic white matter pathways (tractography) did not show any statistically significant association with any of the CSF biomarkers, core clinical features, nor the clinical or cognitive measures within LB patients or healthy controls (*P* > 0.05). The details of these analyses are available in Supplementary Table 3.

## Discussion

In this study, we investigated the association of CHIPS with other MRI measures of global WMSA and cholinergic degeneration, as well as with regional brain atrophy, CSF biomarkers of Alzheimer’s disease co-pathology, core clinical features and cognitive measures in patients within the clinical LB continuum.

We found that LB patients exhibited a significantly higher degree of WMSA in cholinergic white matter compared with controls. In particular, LB patients had significantly more WMSA in both the anterior and posterior regions of the external capsule, but not in the cingulum, as indicated by CHIPS subdomains. The higher CHIPS scores in the external capsule were in line with the higher mean diffusivity in the external capsule as shown by the tractography method. This finding suggests a more pronounced cholinergic degeneration in the external capsule when comparing LB patients with controls. These group differences were independent of the effects of age and education. Regarding mechanistic interpretations, Barber *et al*.^49^ postulated that WMSA in deep white matter such as the external capsule may be related to CVD, whereas WMSA in periventricular white matter could relate to neurodegeneration. Therefore, our findings suggest the preferential vulnerability of cholinergic pathways to CVD co-pathology in the clinical LB continuum and highlight the key role cholinergic degeneration may play in the disease manifestation.^5,50,51^ Recent *in vivo* findings demonstrated severe cholinergic terminal loss in newly diagnosed DLB patients, particularly in limbic and cortical areas, suggesting that cholinergic degeneration is an early and central component of LB pathology.^52^ The cholinergic deficits observed in our study, particularly in the external capsule, align with this finding and suggest that cerebrovascular pathology may further exacerbate cholinergic white matter damage in patients in the LB continuum. Additionally, our findings align with the only previous study using CHIPS in DLB, which also reported significantly higher CHIPS scores in LB patients compared to controls, particularly affecting the external capsule.^19^

Regarding the cingulum cholinergic pathway, tractography analysis exhibited a significantly higher cingulum mean diffusivity in LB compared with controls, independent of the effects of age and education. In contrast, the CHIPS score could not capture any statistically significant difference in cingulum between the two groups. Prior studies suggested that tractography (relying on diffusion tensor imaging) might be more sensitive to subtle white matter changes compared with visual rating methods for WMSA.^53-55^ Tractography may therefore capture early microstructural white matter changes that are not yet detectable by the CHIPS. Our tractography results are in line with the previous studies by Schumacher *et al*.,^13,14^ who showed a significant difference in cingulum mean diffusivity between manifest DLB patients and controls and significant but less prominent differences between MCI-LB and controls. Therefore, in addition to different sensitivities of CHIPS and tractography, cholinergic pathways may not be uniformly affected along the LB continuum, with overt degeneration of the cingulum possibly occurring later in the disease process. PET studies have shown that the cingulate gyrus metabolism is relatively preserved in manifest DLB, further supporting this notion.^56^ The cingulate island sign, which refers to the relative preservation of posterior cingulate metabolism in relation to precuneus and cuneus metabolism, is a supportive biomarker in the diagnostic criteria of DLB.^22^ It has been hypothesized that the preservation of the posterior cingulate in DLB is due to relatively preserved blood perfusion in that area, and neuropathological studies reported low alpha-synuclein-related pathology in the posterior cingulate compared to anterior cingulate in DLB.^57-59^ Given the relatively early stage of the disease in our cohort, we may have only captured changes in the cingulum with the tractography method and not with CHIPS.

Our findings on the relative preservation of cholinergic innervation to the cingulum are further supported by a recent study by Okkels *et al*.,^60^ which suggests that cholinergic basal forebrain degeneration in Lewy body disease follows a structured posterior-to-anterior pattern. The anterior part of the nucleus basalis of Meynert (NBM), which provides cholinergic projections to the cingulum and amygdala, is affected later in disease progression than the posterior and intermediate NBM, which supply the superior temporal gyrus and neocortex, respectively. This pattern indicates that cholinergic loss in cingulum becomes more pronounced as dementia progresses, aligning with our findings.

Our findings suggest that global WMSA (Fazekas and FreeSurfer) reflects age-related changes rather than disease-specific findings, as the differences between LB patients and controls disappeared after adjusting for age (and education). Additionally, the lack of an age-by-diagnosis interaction indicates that global WMSA accumulation in LB does not exceed what is expected for normal aging. Previous studies have shown that age is a significant risk factor for WMSA,^5,61,62^ supporting our interpretation that these changes are largely age-related. In contrast, the CHIPS and cholinergic pathway findings suggest disease-specific white matter alterations in LB, independent of global WMSA. The greater cholinergic WMSA and cholinergic degeneration in LB, despite similar global WMSA levels between groups, suggests that cholinergic pathways may be particularly vulnerable to WMSA in LB, regardless of overall white matter burden.

The ROC analysis showed that CHIPS in the posterior external capsule and tractography measures had the highest AUC values. CHIPS in the posterior external capsule and cingulum mean diffusivity surpassed the 80% sensitivity and specificity threshold for an optimal biomarker.^63^ In contrast, Fazekas and CHIPS in the cingulum had lower AUC values, suggesting they are less effective for discriminating LB from controls, while they may still reflect white matter damage and neurodegeneration.

We also investigated associations among MRI measures to gain deeper mechanistic insights into CHIPS. We found a significant association between the two cholinergic methods, i.e. CHIPS and tractography. This correlation suggests that CHIPS is a reliable clinical method and could facilitate the assessment of WMSA in the cholinergic white matter in clinical routine, especially considering the complexity and challenges of implementing tractography clinically. The fact that the association between CHIPS and tractography was only statistically significant in the LB group but not in controls suggests disease-specific interpretations and highlights clinical implications of the CHIPS. Additionally, we observed an association between both global WMSA methods, i.e. Fazekas scale and FreeSurfer, in LB patients but not in controls. This finding aligns with previous research that reported a correlation between these two methods.^28,30,64^ These analyses on associations align with the group differences discussed above, indicating that our cholinergic findings may be disease-specific in LB patients, while global WMSA findings may partly reflect age effects.

We next investigated the association of CHIPS with MRI markers of neurodegeneration (i.e. MTA, PA and GCA-F scores). Our findings highlight the role of CVD co-pathology in LB neurodegeneration, particularly in frontal atrophy. Higher CHIPS scores were associated with increased frontal atrophy in LB patients but not with medial temporal or posterior atrophy, aligning with previous reports linking global WMSA to frontal cortex atrophy.^5,6^ Our study expands on this by incorporating cholinergic WMSA and tractography, all of which showed associations with frontal atrophy. In contrast, CHIPS scores were not significantly associated with medial temporal atrophy, suggesting that CVD-related cholinergic damage does not primarily drive medial temporal degeneration in LB continuum. Instead, our previous research suggest that both global CVD burden and Alzheimer’s disease co-pathology may contribute to medial temporal atrophy,^6^ which is consistent with previous studies showing that Fazekas-MTA associations were influenced by Alzheimer’s disease pathology.^65^ The lack of a statistically significant association between CHIPS and medial temporal atrophy suggests that CVD alone may not be sufficient to drive medial temporal atrophy in LB and supports the idea that global WMSA burden together with Alzheimer’s disease pathology may play a key role in this process.^6,65^

Overall, these associations were more pronounced in LB patients than in controls, reinforcing the role of CVD in neurodegeneration beyond normal aging effects.

Our study has some limitations. Firstly, the cross-sectional design prevents us from establishing causal relationships between WMSA and the other measures, which would be better addressed in a longitudinal study. Secondly, we utilized well-established MRI markers of CVD, which, though valuable, do not account for other possible complexities of CVD in LB patients and lack complete vascular specificity. Finally, there was a statistically significant difference in age and years of education between LB patients and healthy controls. Therefore, when comparing these two diagnostic groups, adjustments for age and years of education were made to account for their potential effect on the results. When performing within-group analyses, we interpreted as disease-specific those findings that were statistically significant in LB patients but not in controls. Findings that were statistically significant in both groups were clarified with a test for a statistical interaction to demonstrate stronger associations in LB patients going beyond and above those found in the healthy population.

## Conclusion

In our study, cholinergic WMSA were significantly more pronounced in LB patients compared to healthy controls above and beyond global WMSA. Mechanistically, we have demonstrated that CVD in cholinergic white matter may be implicated in frontal atrophy along the LB continuum. In contrast, medial temporal atrophy appears to be more related to global CVD and/or Alzheimer’s disease co-pathology, as suggested in recent studies.^6,65,66^ Clinically, our findings underscore the potential of CHIPS for assessing cholinergic WMSA in clinical settings, thereby aiding in the radiological characterization of LB patients. Altogether, we conclude that CVD co-pathology could be one of the drivers of the well-documented cholinergic degeneration in people with LB disease, highlighting opportunities for therapeutic interventions that target vascular health in the LB continuum, from early stages of MCI-LB extending to full-blown DLB.

## Supplementary Material

fcaf173_Supplementary_Data

## Data Availability

The data and code that support the findings of this study are available from the corresponding author, upon reasonable request.
