# Supplementary material for: Cerebrovascular co-pathology and cholinergic white matter pathways along the Lewy body continuum
Source: Brain Commun. 2025 May 6;7(3):fcaf173. doi: 10.1093/braincomms/fcaf173 (PMC12086334; doi:10.1093/braincomms/fcaf173)
Supplement: fcaf173_Supplementary_Data [file fcaf173_supplementary_data.docx]

**Supplementary material**

**Supplementary Table 1: MRI scanning parameters.**

| **MRI acquisition parameter** | **Structural T1 MRI** | **Structural FLAIR MRI** | **Diffusion-weighted imaging MRI** |
| --- | --- | --- | --- |
| Protocol | MPRAGE | 3D FLAIR | Echo-planar Imaging |
| Repetition Time (ms) | 8.2 | 10000 | 10800 |
| Echo Time (ms) | 3.8 | 140 | 78 |
| Slices (count) | 160 | 140 | 80 |
| Slice thickness (mm) | 1 | 1 | 2 |
| Voxel Size (mm) | 0.94×0.94×1.00 | 0.10×0.90×1.00 | 1.60×1.60×2.00 |
| Specific Parameters | - | - | Single b = 0 image. 32 gradient directions with b = 1000. One inverted phase encoding b = 0 |

MRI = Magnetic Resonance Imaging; MPRAGE = Magnetization-Prepared Rapid Acquisition Gradient Echo; FLAIR = Fluid-attenuated inversion recovery.

**Supplementary Table 2: CHIPS scale regions scoring.**

| **Slices** | **Regions** | **Score** | **Factor** | **Total** |
| --- | --- | --- | --- | --- |
| 1. Low External Capsule | EC anterior | 0-1-2 | 4 | 0-4-8 |
|  | EC posterior | 0-1-2 | 4 | 0-4-8 |
| 2. High External Capsule | Cingulate | 0-1-2 | 4 | 0-4-8 |
|  | EC anterior | 0-1-2 | 3 | 0-3-6 |
|  | EC posterior | 0-1-2 | 3 | 0-3-6 |
| 3. Corona Radiata | Anterior | 0-1-2 | 2 | 0-2-4 |
|  | Posterior | 0-1-2 | 2 | 0-2-4 |
|  | Cingulate | 0-1-2 | 1 | 0-1-2 |
| 4. Centrum Semiovale | Anterior | 0-1-2 | 1 | 0-1-2 |
|  | Posterior | 0-1-2 | 1 | 0-1-2 |

Score is determined using a three-point system (0 = normal; 1 = <50% involvement; 2≥50% involvement). EC = External Capsule.

**Supplementary Table 3: Associations between demographic data, WMSA measures, atrophy measures, CSF biomarkers, cognitive measures and core clinical features expressed in p-values with effect sizes expressed as Cohen’s d.**

|  | **Lewy body group** | | | | | | | | | | **Healthy controls** | | | | | | | | | |
| --- | --- | --- | --- | --- | --- | --- | --- | --- | --- | --- | --- | --- | --- | --- | --- | --- | --- | --- | --- | --- |
|  | CHIPS | | Fazekas | | FreeSurfer WMSA | | MD External capsule | | MD Cingulum | | CHIPS | | Fazekas | | FreeSurfer WMSA | | MD External capsule | | MD Cingulum | |
|  | p | d | p | d | p | d | p | d | p | d | p | d | p | d | p | d | p | d | p | d |
| Visual Hallucinations | 0.957 | 0.017 | 0.202 | 0.199 | 0.463 | -0.252 | 0.964 | 0.016 | 0.603 | -0.174 | - | - | - | - | - | - | - | - | - | - |
| RBD | 0.180 | -0.437 | 0.249 | 0.180 | 0.906 | -0.001 | 0.942 | 0.024 | 0.933 | -0.028 | - | - | - | - | - | - | - | - | - | - |
| Parkinsonism | - | - | 0.418 | 0.126 | 0.509 | - | - | - | - | - | - | - | - | - | - | - | - | - | - | - |
| Cognitive Fluctuations | 0.606 | 0.196 | 0.706 | 0.059 | 0.688 | -0.193 | 0.046 | -0.867 | 0.052 | -0.843 | - | - | - | - | - | - | - | - | - | - |
| DAT scan | 0.192 | -0.625 | 0.508 | 0.130 | 0.533 | 0.007 | 0.969 | 0.018 | 0.875 | 0.075 | - | - | - | - | - | - | - | - | - | - |
| Mini-mental state examination | 0.434 | -0.494 | 0.967 | 0.000 | 0.382 | -0.886 | 0.224 | 9.890 | 0.099 | 9.890 | 0.226 | 2.871 | 0.059 | 0.652 | 0.470 | 0.731 | 0.981 | 39.378 | 0.859 | 38.827 |
| Unified Parkinson's disease rating scale | 0.069 | -0.916 | 0.352 | -0.336 | 0.044 | 2.106 | 0.204 | 2.425 | 0.319 | 2.425 | - | - | - | - | - | - | - | - | - | - |
| Geriatric depression scale | 0.668 | -1.572 | 0.324 | 0.332 | 0.508 | -0.669 | 0.044 | 2.594 | 0.077 | 2.594 | - | - | - | - | - | - | - | - | - | - |
| Global deterioration scale | 0.367 | -2.467 | 0.222 | -0.391 | 0.516 | 0.656 | 0.016 | 7.633 | 0.011 | 7.633 | - | - | - | - | - | - | - | - | - | - |
| Boston naming Test | 0.062 | 1.097 | 0.578 | 0.190 | 0.087 | -1.763 | 0.415 | 8.049 | 0.453 | 8.049 | 0.106 | 6.527 | 0.138 | 0.580 | 0.959 | -0.052 | 0.526 | 23.087 | 0.600 | 22.765 |
| FCSRT total free recall | 0.652 | -1.606 | 0.840 | -0.033 | 0.814 | 0.238 | 0.207 | 1.750 | 0.128 | 1.750 | 0.110 | 2.208 | 0.304 | 0.369 | 0.164 | -1.421 | 0.044 | 6.084 | 0.059 | 5.999 |
| FCSRT total recall | 0.739 | -0.283 | 0.687 | -0.107 | 0.948 | -0.066 | 0.424 | 2.740 | 0.284 | 2.740 | 0.512 | 4.904 | 0.376 | -0.401 | 0.763 | 0.304 | 0.083 | 17.934 | 0.207 | 17.683 |
| Digits span - direct | 0.587 | -2.321 | 0.888 | 0.011 | 0.554 | -0.597 | 0.233 | 6.109 | 0.492 | 6.109 | 0.722 | -0.703 | 0.263 | -0.459 | 0.260 | -1.144 | 0.392 | 8.267 | 0.088 | 8.152 |
| Digits span - reverse | 0.764 | -2.437 | 0.081 | 0.657 | 0.183 | -1.359 | 0.952 | 4.490 | 0.669 | 4.490 | 0.691 | -0.835 | 0.213 | 0.224 | 0.586 | -0.549 | 0.252 | 6.158 | 0.135 | 6.072 |
| Semantic fluency | 0.701 | -1.667 | 0.822 | 0.075 | 0.079 | -1.812 | 0.504 | 4.050 | 0.420 | 4.050 | 0.643 | 1.356 | 0.626 | 0.174 | 0.583 | -0.555 | 0.372 | 6.926 | 0.259 | 6.830 |
| Phonetic fluency | 0.230 | -1.981 | 0.187 | 0.444 | 0.313 | -1.025 | 0.246 | 2.872 | 0.102 | 2.872 | 0.166 | 0.786 | 0.148 | 0.523 | 0.463 | -0.742 | 0.478 | 4.625 | 0.588 | 4.560 |
| Visual objects and space perception battery | 0.215 | -2.119 | 0.425 | 0.214 | 0.055 | -1.989 | 0.691 | 3.271 | 0.315 | 3.271 | 0.118 | -0.110 | 0.770 | 0.050 | 0.877 | 0.156 | 0.454 | 10.683 | 0.306 | 10.534 |
| Neuropsychiatric inventory total score | 0.855 | -0.971 | 0.486 | -0.285 | 0.047 | 2.105 | 0.028 | 1.741 | 0.017 | 1.741 | 0.842 | -1.531 | 0.774 | 0.070 | 0.124 | 1.594 | 0.280 | 0.617 | 0.719 | 0.605 |
| Clinical dementia rate | 0.730 | -2.667 | 0.786 | -0.245 | 0.341 | 0.965 | 0.137 | 2.364 | 0.037 | 2.365 | 0.378 | -1.601 | 0.614 | 0.215 | 0.892 | -0.137 | 0.277 | 0.231 | 0.254 | 0.231 |
| AB42-40 ratio | 0.520 | -2.835 | 0.230 | -0.372 | 0.647 | 0.462 | 0.743 | 3.392 | 0.776 | 3.402 | 0.029 | -1.518 | 0.170 | 0.283 | 0.275 | -1.115 | 0.448 | 0.920 | 0.373 | 0.905 |
| Total tau | 0.705 | 2.485 | 0.328 | 0.283 | 0.937 | -0.079 | 0.863 | 2.735 | 0.811 | 2.735 | 0.467 | 1.935 | 0.220 | 0.361 | 0.879 | 0.154 | 0.572 | 3.424 | 0.923 | 3.362 |
| Phosphorylated tau | 0.701 | 1.270 | 0.264 | 0.258 | 0.986 | 0.017 | 0.555 | 2.256 | 0.517 | 2.256 | 0.562 | 1.144 | 0.157 | 0.326 | 0.942 | 0.073 | 0.527 | 2.888 | 0.821 | 2.836 |

P-values and Cohen’s d values are included for variable pairs. Effect sizes were expressed as Cohen’s d to allow for direct comparison across statistical tests. Statistical significance was set at P < .05 for all analyses. p = p-value, d = Cohen’s d, RBD = REM sleep behavior disorder, FCSRT = Free and Cued Selective Reminding Test, AB40-42 = Amyloid-beta 40-42.

**Supplementary Table 4: ROC analysis results for discriminating DLB from HC, including AUC values, confidence intervals, optimal cutoff values, sensitivity, and specificity for WMSA and tractography measures (ordered by AUC values).**

| **WMSA measure** | **AUC** | **p-value** | **95% Confidence Interval** | | **Cutoff*** | | **Sensitivity** | **Specificity** |
| --- | --- | --- | --- | --- | --- | --- | --- | --- |
|  |  |  | Lower Bound | Upper Bound | |  |  |  |
| CHIPS Total posterior excap | 0.887 | <0.001 | 0.808 | 0.966 | | 6 | 0.806 | 0.833 |
| MD Cingulum | 0.873 | <0.001 | 0.793 | 0.954 | | 0.000987 | 0.806 | 0.806 |
| MD External Capsule | 0.873 | <0.001 | 0.793 | 0.953 | | 0.0011915 | 0.778 | 0.833 |
| CHIPS Total excap | 0.868 | <0.001 | 0.786 | 0.949 | | 18.5 | 0.722 | 0.889 |
| CHIPS Total | 0.861 | <0.001 | 0.778 | 0.945 | | 20 | 0.722 | 0.917 |
| FreeSurfer WMSA | 0.81 | <0.001 | 0.708 | 0.912 | | 2095.75 | 0.75 | 0.778 |
| CHIPS Total anterior excap | 0.797 | <0.001 | 0.695 | 0.899 | | 13.5 | 0.583 | 0.889 |
| Fazekas Low/High | 0.667 | 0.015 | 0.54 | 0.793 | | 0.5 | 0.611 | 0.722 |
| CHIPS Cingulum | 0.652 | 0.026 | 0.525 | 0.78 | | 0.5 | 0.472 | 0.806 |

Excap = External capsule; CHIPS = Cholinergic pathways hyperintensities scale; WMSA = White matter signal abnormalities; AUC = Area under curve; *Equals to or higher means positive outcome.
